# Osseointegration Potential Assessment of Bone Graft Materials Loaded with Mesenchymal Stem Cells in Peri-Implant Bone Defects

**DOI:** 10.3390/ijms25020862

**Published:** 2024-01-10

**Authors:** Kuo-Fang Tseng, Shiau-Ting Shiu, Chia-Yi Hung, Ya-Hui Chan, Tze-Jian Chee, Pai-Chun Huang, Pin-Chuang Lai, Sheng-Wei Feng

**Affiliations:** 1 School of Dentistry, College of Oral Medicine, Taipei Medical University, Taipei City 110301, Taiwan; 2 Department of Dentistry, Shuang Ho Hospital, Taipei Medical University, New Taipei City 235041, Taiwan; 3 School of Dentistry and Graduate Institute of Dental Science, National Defense Medical Center, Taipei City 114201, Taiwan; 4 School of Oral Hygiene, College of Oral Medicine, Taipei Medical University, Taipei City 110301, Taiwan; 5 Department of Periodontics, School of Dentistry, University of Missouri-Kansas City, Kansas City, MO 64108, USA; 6 Division of Prosthodontics, Department of Dentistry, Taipei Medical University Hospital, Taipei City 11031, Taiwan

**Keywords:** implant stability, osseointegration, peri-implant defect, bone graft materials, mesenchymal stem cells

## Abstract

Many studies have been exploring the use of bone graft materials (BGMs) and mesenchymal stem cells in bone defect reconstruction. However, the regeneration potential of Algipore (highly purified hydroxyapatite) and Biphasic (hydroxyapatite/beta-tricalcium phosphate) BGMs combined with bone marrow–derived mesenchymal stem cells (BMSCs) remains unclear. Therefore, we evaluated their osseointegration capacities in reconstructing peri-implant bone defects. The cellular characteristics of BMSCs and the material properties of Algipore and Biphasic were assessed in vitro. Four experimental groups—Algipore, Biphasic, Algipore+BMSCs, and Biphasic+BMSCs—were designed in a rabbit tibia peri-implant defect model. Implant stability parameters were measured. After 4 and 8 weeks of healing, all samples were evaluated using micro-CT, histological, and histomorphometric analysis. In the energy-dispersive X-ray spectroscopy experiment, the Ca/P ratio was higher for Algipore (1.67) than for Biphasic (1.44). The ISQ values continuously increased, and the PTV values gradually decreased for all groups during the healing period. Both Algipore and Biphasic BGM promoted new bone regeneration. Higher implant stability and bone volume density were observed when Algipore and Biphasic BGMs were combined with BMSCs. Biphasic BGM exhibited a faster degradation rate than Algipore BGM. Notably, after eight weeks of healing, Algipore with BSMCs showed more bone–implant contact than Biphasic alone (*p* < 0.05). Both Algipore and Biphasic are efficient in reconstructing peri-implant bone defects. In addition, Algipore BGM incorporation with BSMCs displayed the best performance in enhancing implant stability and osseointegration potential.

## 1. Introduction

Immediate implantation into fresh extraction sockets is a common clinical therapeutic approach, with advantages such as short treatment time, few surgical procedures, and favorable alveolar ridge preservation [1,2]. This technique has osseointegration performance and success rates comparable to implants on healed sites [3]. However, the procedure creates surgical bone defects between the dental implants and extraction sockets. The size and shape discrepancies between dental implants and extraction sockets determine the dimensions of the peri-implant defects. Among the defect types, circumferential bone defects are the most difficult for tissue regeneration and clinical management [4].

The main challenges of circumferential peri-implant bone defects are the lack of primary implant stability, intact wound closure, and long-term bone tissue prognosis. Moreover, the potential benefits of bone grafts for peri-implant bone defects remain controversial [5,6,7,8,9,10]. Still, the selection of bone graft material (BGM) is crucial for the long-term success of osseointegration. Immediate implant placement can provide adequate implant stability by applying BGM in peri-implant bone defects [1,11]. Autogenous bone is the gold-standard BGM because of its superior osteoconductive, osteoinductive, and osteogenic properties [12]. To avoid additional surgical trauma and the risk of disease transmission, synthetic and nature-derived BGM, such as hydroxyapatite and beta-tricalcium phosphate, can also be used [13,14,15]. These BGMs possess high biocompatibility, adequate mechanical properties, and favorable osteoconductive properties, whereas the lack of vascularization and osteoinduction properties limit their biological performance [12].

Among these BGMs, Symbios Algipore BGM (Symbios^®^ Algipore, AlgOss Biotechnologies GmbH, Vienna, Austria) consists of almost 100% highly porous hydroxyapatite derived from red marine algae. The columnar structure with interconnective pores in the 5–10 μm range and gradual resorption kinetics provides a large surface area for protein adsorption and a suitable surface as growth factor or stem cell carrier [16]. In addition, Symbios Biphasic BGM (Symbios^®^ Biphasic BGM, AlgOss Biotechnologies GmbH, Vienna, Austria) is a modification of Algipore BGM, composed of 20% hydroxyapatite (Algipore BGM) and 80% beta-tricalcium phosphate. The honeycomb-like tubular structure and interconnected porous surface with a faster resorption rate are suitable for tissue ingrowth and bone deposition [17]. Both Algipore and Biphasic BGMs have been demonstrated to promote bone formation in preclinical experiments and clinical studies [18,19,20].

Scaffolds, cells, and molecular elements are used or assembled for therapeutic applications in bone tissue engineering. Mesenchymal stem cells (MSCs) are undifferentiated cells with high self-renewal capacity, multipotent differentiation potential, and immunomodulatory and regenerative properties, making them valuable for research and clinical application [21,22,23]. Many studies have evaluated the use of BGMs combined with bone marrow-derived MSCs (BMSCs) in the reconstruction of bone defects and osseointegration enhancement [24,25,26].

Although studies have compared different surface treatments of dental implants and BGMs [3,4,27,28], few have evaluated the synergistic effects of BGM and BMSCs on the bone regeneration of surgically created peri-implant bone defects [29,30]. Therefore, we evaluated the bone regeneration capacities of algae-derived plant hydroxyapatite (Algipore) and hydroxyapatite/beta-tricalcium phosphate (Biphasic) combined with BMSCs for reconstructing circumferential bone defects of modern dental implants in a rabbit tibia model. In addition, implant stability parameters, micro-CT analysis, and histological evaluation were performed to compare the mechanical and biological efficiency of the combination of BGM and BMSCs.

## 2. Results

### 2.1. BMSC Isolation, Culture, and Osteogenic Differentiation

**Figure 1:**
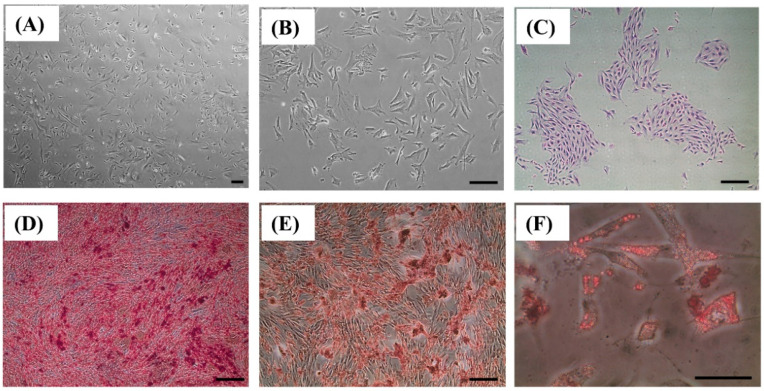
Identification and characteristics of bone marrow mesenchymal stem cells (BMSCs). (**A**,**B**) Representative microscopic illustrations of BMSCs (P5, Original magnification ×10 and ×40). (**C**) Representative images of colony-forming units of BMSCs. (**D**) Alizarin red staining of calcium deposits indicates the osteogenic capacities of BMSCs. (**E**) Safranin O staining of the cartilage matrix indicates the chondrogenic abilities of BMSCs. (**F**) Oil Red O staining of intracellular lipid accumulation indicates adipogenic capacities of BMSCs. Scale bar = 100 μm.

The BMSCs obtained from the bone marrow of rabbits exhibited homogenous, spindle-shaped, and fibroblast-like morphology (Figure 1A–C), as well as multilineage differentiation potential. After 14 days of osteogenic induction, BMSCs formed abundant extracellular calcium deposition, as demonstrated using Alizarin red staining (Figure 1D). After 14 days of chondrogenic induction, BMSCs formed a chondrogenic matrix and proteoglycan deposition, as shown using Safranin O staining (Figure 1E). After 14 days of adipogenic induction, BMSCs formed lipid droplets, as demonstrated using Oil red O staining (Figure 1F).

### 2.2. SEM and EDS

**Figure 2:**
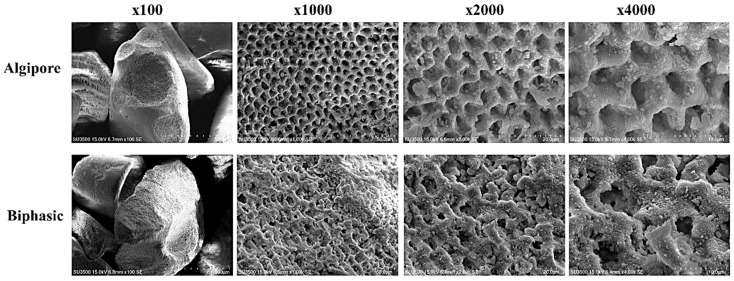
Scanning electron microscopic images of the porous structure of Algipore and Biphasic bone graft materials at different magnifications (×100, ×1000, ×2000, ×4000).

SEM images of Algipore and Biphasic BGMs displayed irregular massive granule types without any contamination (Figure 2). At higher magnification (×1000, ×2000, and ×4000), homogeneous microporous structures and interconnected pores were observed on the surface of Algipore and Biphasic BGMs. Algipore BGMs showed a regular columnar structure, whereas Biphasic BGMs showed an irregular coral-like form (Figure 2).

**Figure 3:**
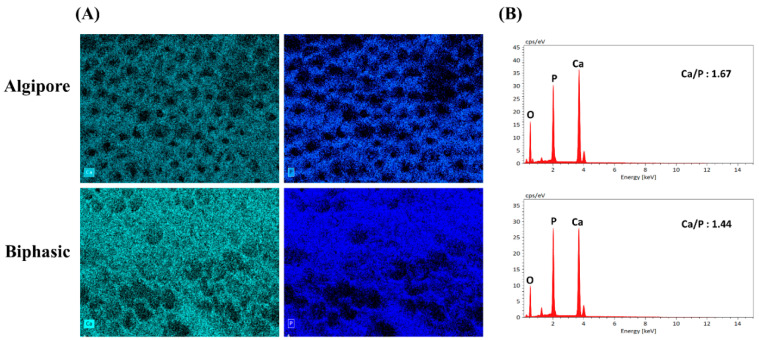
Energy-dispersive X-ray spectroscopy (EDS) images of Algipore and Biphasic bone graft materials. (**A**) EDS elemental mapping of Algipore and Biphasic for calcium and phosphate elements, respectively. (**B**) The EDS spectrum and Ca/P ratio of Algipore and Biphasic bone graft materials.

The element composition of Algipore and Biphasic BGMs was assessed using EDS (Figure 3). In Figure 3A, calcium, and phosphate elements are mapped in green and blue, respectively, on the surfaces of both Algipore and Biphasic BGMs. In addition, a higher Ca/P ratio was observed in Algipore BGMs (1.67) than in Biphasic BGMs (1.44) (Figure 3B).

### 2.3. Animal Status and Implant Surgery Result

**Figure 4:**
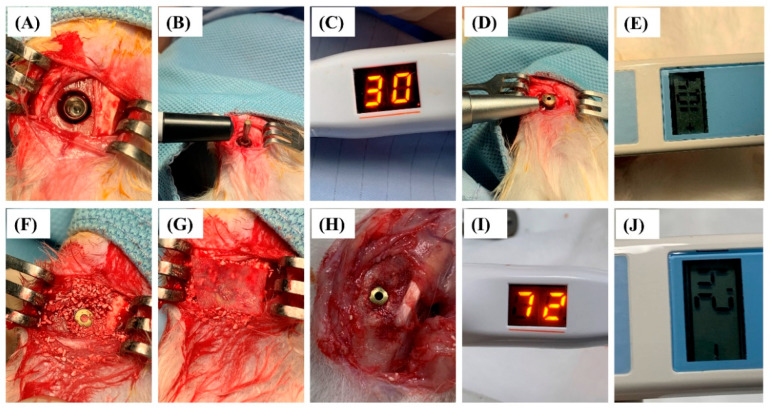
Surgical procedures and implant stability detection at surgery and after 4 and 8 weeks of healing. (**A**) Creation of circumferential peri-implant bone defects. (**B**) The Penguin (resonance frequency analyzer) was used to measure the ISQ values immediately after implant placement. (**C**) Lower ISQ values (30) were demonstrated. (**D**) Periotest M was used to measure the PTV values immediately after implant placement. (**E**) Higher PTV values (10.4) were observed. (**F**) Algipore and Biphasic bone graft materials combined with mesenchymal stem cells were placed in the bone defect area. (**G**) The collagen membrane was covered on the implant and bone graft materials. (**H**) Well-healing status was observed after four weeks of healing. (**I**) Higher ISQ values (72) were demonstrated after four weeks of healing. (**J**) Better implant stability was also demonstrated by Periotest M with −2.4 PTV values after four weeks of healing.

Regarding the health status, all experimental rabbits recovered well postoperatively and during the experimental phase. As presented in Figure 4H, all implants were maintained and well-integrated into the bone without complications.

### 2.4. Implant Stability Detection

**Figure 5:**
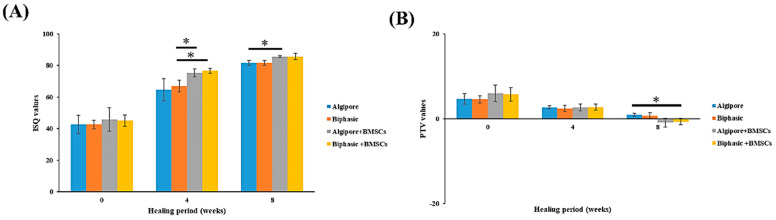
Implant stability parameters at surgery and after 4 and 8 weeks of healing. (**A**) ISQ and (**B**) PTV values of implants of the Algipore, Biphasic, Algipore+BMSCs, and Biphasic+BMSCs groups at multiple experimental times. The ISQ values continuously increased for all groups during the healing period, whereas the PTV values gradually decreased. (* *p* < 0.05).

Implant stability was evaluated using ISQ values obtained from the resonance frequency analyzer and PTV values from the Periotest M device. As illustrated in Figure 5A, the ISQ values in all four groups gradually increased during the entire healing period. The initial ISQ values detected immediately after implant insertion for the Algipore, Biphasic, Algipore+BMSCs, and Biphasic+BMSCs groups were 42.6 ± 5.9, 42.6 ± 2.7, 45.7 ± 7.4, and 45 ± 3.56, respectively. After 4 weeks of healing, the Algipore+BMSCs (75.3 ± 2.5) and Biphasic+BMSCs (76.6 ± 1.5) groups exhibited significantly higher ISQ values than the Biphasic group (67 ± 3.6; *p* < 0.05). At 8 weeks, a significant difference was observed only between the Algipore (81.6 ± 1.5) and Algipore+BMSCs groups (85.6 ± 0.5; *p* < 0.05).

The PTV values measured immediately after implant insertion were 4.6 ± 1.2, 4.5 ± 0.8, 6.0 ± 1.9, and 5.7 ± 1.5 in the Algipore, Biphasic, Algipore+BMSCs, and Biphasic+BMSCs groups, respectively (Figure 5B). At 4 weeks, all groups exhibited PTV values that decreased by approximately 50% compared with baseline PTV values, but the differences were not significant. At 8 weeks, the Biphasic+BMSCs (−0.7 ± 0.7) group exhibited significantly lower PTV values than the Algipore group (0.9 ± 0.40; *p* < 0.05).

### 2.5. Micro-CT Measurements

**Figure 6:**
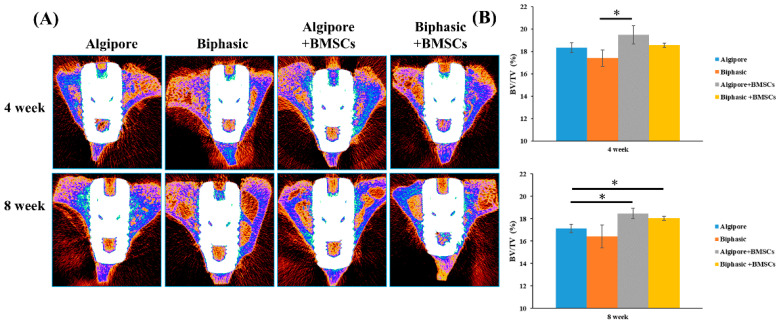
Micro-CT analysis of osseointegration performance among the Algipore, Biphasic, Algipore+BMSCs, and Biphasic+BMSCs groups 4 and 8 weeks after surgery. (**A**) 2D-reconstructed CT images of implants and the surrounding bone structures at 4 and 8 weeks. (**B**) Quantitative analysis of the percentages of bone volume/total volume (BV/TV%) (* *p* < 0.05).

The micro-CT two-dimensional reconstruction images of the cross-sections of the implant/bone complex at eight weeks are presented in Figure 6A. The BV density (BV/TV%) within the VOI for the four groups after 4 and 8 weeks is shown in Figure 6B. At four weeks, BV/TV values were higher in the Algipore+BMSCs (19.5% ± 0.8%) and Biphasic+BMSCs (18.5% ± 0.1%) groups than in the Algipore (18.3% ± 0.4%) and Biphasic (17.4% ± 0.7%) groups after four weeks of healing; however, the difference was significant only between the Biphasic and Algipore+BMSCs groups. At 8 weeks, the Algipore+BMSCs (18.4% ± 0.4%) and Biphasic+BMSCs (18% ± 0.1%) groups exhibited significantly higher BV/TV values than the Algipore group (17.1% ± 0.3%, *p* < 0.05).

### 2.6. Histological and Histomorphometric Analyses

**Figure 7:**
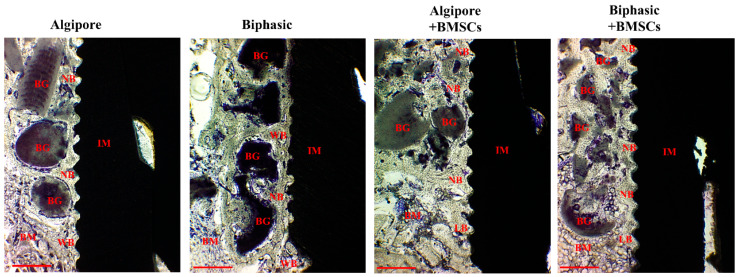
Histological analysis of newly formed bone around bone graft materials and implants among Algipore, Biphasic, Algipore+BMSCs, and Biphasic+BMSCs groups four weeks after surgery. Toluidine blue stain, bone graft (BG), new bone (NB), woven bone (WB), lamellar bone (LB), implant (IM), and bone marrow (BM). Original magnification ×40. Scale bar: 500 μm.

Histological evaluation revealed well-integrated implants in all experimental groups at the end of 4 and 8 weeks. At four weeks, in all experimental groups, the Algipore and Biphasic bone grafts were observed around the implants (Figure 7). They were surrounded by irregular and newly formed bone, with some bone grafts contacting the implant surface. The grafts also had more intimate contact with the newly formed bone when incorporated with BMSCs. The implants of the four groups were well-integrated with the woven bone and some parallel lamellar bone. More woven bone was demonstrated in the Algipore+BMSCs and Biphasic+BMSCs groups, with more extension to the bone marrow area, than in the Algipore and Biphasic groups.

**Figure 8:**
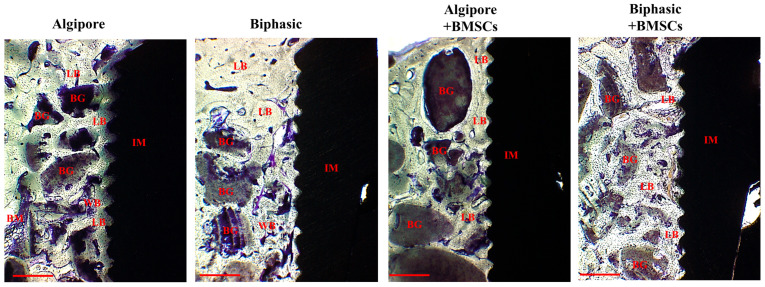
Histological analysis of newly formed bone around bone graft materials and implants among Algipore, Biphasic, Algipore+BMSCs, and Biphasic+BMSCs groups eight weeks after surgery. Toluidine blue stain, bone graft (BG), new bone (NB), woven bone (WB), lamellar bone (LB), implant (IM), and bone marrow (BM). Original magnification ×40. Scale bar: 500 μm.

At eight weeks, Algipore and Biphasic bone grafts had intimate contact with compact and lamellar bone, and some bone grafts were replaced with new bone formation (Figure 8). Biphasic bone grafts exhibited more resorption and degradation than Algipore bone grafts. The four groups’ implant surface and thread areas were all well-integrated and wholly occupied with mature lamellar bone, especially in the Algipore+BMSCs and Biphasic+BMSCs groups.

**Figure 9:**
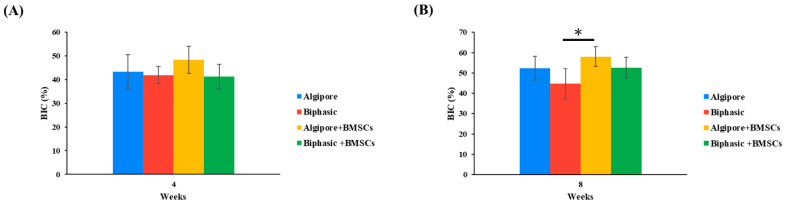
Histomorphometric analysis of direct bone contact values with implants (BIC). Mean BIC values (±SD) for the Algipore, Biphasic, Algipore+BMSCs, and Biphasic+BMSCs groups at (**A**) 4 and (**B**) 8 weeks after surgery (* *p* < 0.05).

BIC was demonstrated from the implants’ direct contact with the surrounding bone in the tibia of the rabbits. Figure 9 presents BIC’s mean and standard deviation in the four groups at 4 and 8 weeks. Generally, all groups’ BIC values increased from 4 to 8 weeks. At 4 weeks, no significant difference was noted among the groups. At 8 weeks, the BIC values in the Algipore+BMSCs group (57.9% ± 5%) were significantly higher than those in the Biphasic group (44.7% ± 5.8%; *p* < 0.05), but not in the Algipore (52.3% ± 7.4%) and Biphasic+BMSCs (52.5% ± 4.8%) groups.

## 3. Discussion

An increasing number of clinical practices are currently relevant to immediate implant placement and immediate loading [17]. A consensus has yet to be reached regarding using BGMs in the created bone defects of modern dental implants. Moreover, the regeneration potential of Algipore and Biphasic BGMs combined with BMSCs for reconstructing severe circumferential bone defects remains unknown. Our study is the first to demonstrate that the incorporation of BMSCs into Algipore and Biphasic BGM can enhance bone regeneration capacity in the circumferential peri-implant bone defects, as demonstrated using implant stability parameters, micro-CT scans, and histological and histomorphometric analyses.

The selection of BGM is critical for osseointegration enhancement of peri-implant bone defects. The porous structures, pore sizes, and surface characteristics of BGM play essential roles in enhancing new bone formation because they can provide 3D support for tissue regeneration, enable vascularization, and promote migration, proliferation, and differentiation of osteoblasts and MSCs [31]. In our study, Algipore BGM (pure HA, particle size: 0.5–1.0 mm, pore size: 5–10 μm) exhibited gradual resorption, whereas Biphasic BGM (80% β-TCP and 20% HA, particle size: 0.2–1.0 mm, pore size: 5–10 μm) exhibited moderate resorption. Our results demonstrated that both Algipore and Biphasic BGMs showed similar porous structures and pore sizes (Figure 2). The pore sizes of the Algipore and Biphasic BGM surfaces facilitate cell attachment and bone ingrowth. Similar results have been demonstrated in animal and clinical studies [16,32,33]. Notably, in this study, Algipore BGM exhibited a higher Ca/P ratio (1.67) than Biphasic BGM (1.44) in the EDS experiment (Figure 3), which is similar to the ratio of human bone mineral (Ca/P ratio of 1.55–1.75) [12]. In a previous study, Ca/P ratios of 1.3–1.5 were observed in the medium mineralized bone area and 1.3–1.8 in the high mineralized bone area from the peri-implant human bone [34]. These features demonstrate that Algipore and Biphasic BGMs are efficient scaffolds for promoting early matrix mineralization and bone healing.

As illustrated in Figs. 5, 6, and 9, our study is the first to demonstrate that both Algipore and Biphasic BGMs can enhance implant stability and promote bone regeneration of peri-implant bone defects during the healing process. One animal study revealed a contact percentage of 71% between newly formed bone and Algipore BGM and 33% of residual Algipore particles with a slowly resorbed rate in rabbit tibia defects [32]. Clinical studies have confirmed the osteoconductivity and resorbability of Algipore and Biphasic BGMs in sinus floor augmentation and peri-implant defect grafting [16,17,18,19,33]. Galindo-Moreno et al. (2020) reported that the combination of autogenous bone and Biphasic BGM induced higher vertical resorption and graft collapse than autogenous bone and an organic bovine bone in maxillary sinus augmentation, likely because of a higher remodeling rate, a higher immune response, and the presence of osteoprogenitors [19]. Applying Biphasic BGM in grafting defects of immediate implant placement in molar extraction sites showed comparable results with autogenous bone regarding horizontal bone resorption, implant success rates, and marginal bone levels [17]. Sokolowski et al. (2020) designed a clinical trial to histologically compare Algipore and Biphasic BGMs for sinus floor augmentation [33]. After six months of healing, more residual graft materials and new bone in contact with the graft were noticed in Algipore BGMs, and more new bone formation was observed in Biphasic BGMs. This may be due to the higher resorption rate of Biphasic BGMs [16].

Our histological findings also noted a similar tendency between the Algipore and Biphasic BGM groups. As illustrated in Figs. 7 and 8, BGMs promoted bone regeneration and osseointegration in peri-implant bone defects. After eight weeks of healing, a more intact outline form and more intimate contact with surrounding new bone were demonstrated in Algipore BGM. In contrast, a faster degradation rate, remodeling, and bone ingrowth into BGM were observed in Biphasic BGM at eight weeks compared to the histological findings at four weeks. Although both BGMs are from the same source, the chemical composition, surface characteristics, and the higher resorption of the β-TCP in Biphasic BGM can explain these differences. Therefore, using BGM in peri-implant bone defects can improve osseointegration and increase BIC. Moreover, BGM can facilitate blood clot stabilization and support osteoprogenitor cell invasion and access to the implant surface [35]. The osteoconductive materials can act as a supportive scaffold and an ideal carrier of BMSCs.

Our data also indicated that the incorporation of Algipore and Biphasic BGMs with BMSCs provided higher implant stability and BV/TV% and BIC values than the Algipore and Biphasic BGMs alone (Figure 5, Figure 6 and Figure 9), with the Algipore+BMSCs group exhibiting the best performance. This may be because Algipore BGM is highly analogous to the hydroxyapatite of natural bone and has a large surface area available for protein binding and amino acid adsorption; thus, it is highly suitable as a BMSCs carrier [16]. In the literature, BMSCs are the most commonly used stem cells for reconstructing peri-implant bone defects [4,24,30,36]. Moreover, bone regeneration and osseointegration can be achieved by two distinct mechanisms: contact osteogenesis (the formation of bone directly on the implant surface) and distance osteogenesis (the formation of bone from the old bone surface) [37,38]. Our results revealed that both contact and distance osteogenesis for osseointegration can be accelerated by BGM incorporation with BMSCs. BMSCs can directly proliferate or differentiate into osteoblasts and indirectly secrete bioactive soluble factors or extracellular vesicles through paracrine function [21]. After implantation in peri-implant bone defects, BGM incorporated with BMSCs and the newly generated tissue matrix were reorganized as bone mimetic niches, which is favorable for creating an osteoinduction-like environment to improve osteoblast function and ensure robust bone healing. Future studies should elucidate the underlying mechanisms of the combination.

This rabbit tibial model may not fully replicate human bone biology. The artificially created peri-implant bone defects cannot be directly simulated to the immediate implant placement in the alveolar bone socket, where bone remodeling and turnover differ [27,28,30]. In addition, using commercial bone grafting materials as BMSCs carriers and bone augmentation in peri-implant bone defects is also one of the limitations of the present study. The development and fabrication of multifunctional composite scaffolds or intelligent materials are more critical for comprehensive bone tissue engineering and maximum therapeutic potential. Another limitation is the lack of the seeding efficiency measurement of BMSCs on the Algipore and Biphasic bone grafting materials. In the future study, a well-designed and optimized seeding efficiency experiment is needed.

## 4. Materials and Methods

This study was approved by the Institutional Animal Care and Use Committee of Taipei Medical University, Taipei, Taiwan (approval no. LAC-2017-0126). Twenty-two 6-month-old, male, New Zealand white rabbits weighing between 3.0 and 3.8 kg were used in this study. Two rabbits were used for BMSCs isolation and 20 for implant placement. All rabbits were housed in separate cages at 25 °C under a 12-h light/dark cycle and were monitored daily for general health and food intake for two weeks before the operation. All rabbits survived without any significant impairment or pathology in their public health. The Animal Research conducted this study: Reporting of In Vivo Experiments (ARRIVE) protocol guidelines [39].

### 4.1. BMSC Isolation, Culture, and Characterization

Mononuclear cell populations from rabbit bone marrow were isolated through density gradient centrifugation at 400× *g* for 30 min using Ficoll-Hypaque-Plus solution (GE Healthcare Bio-Sciences, Piscataway, NJ, USA) according to a previously described method [25,26]. The mononuclear cell layer was collected, washed twice with α-MEM, and immediately seeded in a 25-cm^2^ flask containing α-MEM (Gibco, Invitrogen, Grand Island, NY, USA) supplemented with 10% fetal bovine serum (Gibco), 100 U/mL penicillin (Invitrogen), 100 μg/mL streptomycin (Invitrogen), and 0.5% L-ascorbic acid 2-phosphate. Cultures were maintained at 37 °C in incubators containing 5% CO_2_, and the medium was changed every three days. When cells reached 80% confluence, they were passaged. Cells at passages 2–8 were used in the experiments to ensure stem cell quality. After the collected cells were induced with osteogenic, chondrogenic, and adipogenic media for 14 days, the multilineage differentiation capacities of BMSCs were confirmed. Furthermore, the colony-forming unit (CFU) efficiency of BMSCs was evaluated.

### 4.2. Scanning Electron Microscopy and Energy-Dispersive X-ray Spectroscopy

The surface characterization of the Algipore bone graft and Biphasic bone graft was conducted using scanning electron microscopy (SEM) (Hitachi SU-3500, Hitachi High-Technologies, Minato-ku, Tokyo, Japan) at an accelerating 15 kV. The samples were dehydrated, mounted on aluminum stubs, and sputter-coated with a thin layer of gold at 10 kV. In addition, the element composition of the samples was evaluated through energy-dispersive X-ray spectroscopy (EDS) (FE-SEM, Hitachi High-Technologies, Tokyo, Japan). The samples were scanned at a 10-mm working distance, with an acceleration voltage of 20 kV and a size of 512 pixels without any coating material. The elements were detected and analyzed in the EDS spectra. Calcium (Ca) and phosphorus (P) ratios were calculated.

### 4.3. Implants, BGM, and Membranes

Titanium–aluminum–vanadium (Ti6Al4V) implants (diameter: 4.2 mm and length: 8 mm; OsseoSpeed EV, Astra Tech Implant System, Dentsply Sirona Implants, Mölndal, Sweden) were manufactured by CNC Machining Services and were grit-blasted and acid-blasted following the manufacturer’s instructions. A cover screw (OsseoSpeed EV, Astra Tech Implant System, Dentsply Sirona Implants, Mölndal, Sweden) was used after implant placement. In addition, Algipore (pure HA, particle size: 0.5–1.0 mm, volume: 2 mL) (Symbios^®^ Algipore, AlgOss Biotechnologies GmbH, Vienna, Austria) and Biphasic (80% β-TCP and 20% HA, particle size: 0.2–1.0 mm, volume: 1 mL) (Symbios^®^ Biphasic BGM, AlgOss Biotechnologies GmbH, Vienna, Austria) were used as BGMs. The Symbios Collagen Membrane pre-hydrated (20 mm × 30 mm) (Symbios Collagen Membrane pre-hydrated, Collagen Matrix Inc., Paramus, NJ, USA) derived from the bovine pericardium was also applied as a biological barrier.

### 4.4. Surgical Procedures for Implants

All the surgical procedures were performed under sterile conditions. The rabbits were anesthetized intramuscularly with 15 mg/kg tiletamine-zolazepam (Zoletil 50, Virbac, Carros Cedex, France). A disinfectant (beta-iodine antiseptic) was applied to the surgical area. Before the operation, local anesthesia was administered by injecting 1.8 mL of 2% lidocaine (1:100,000 epinephrine). Skin incision, flap preparation, muscle dissection, and periosteal elevation were performed to expose the proximal tibial metaphysis. Subsequently, a physio dispenser and dental handpiece were achieved with torque-reduction rotary instruments (within 40 N as the maximum torque) at 1200 rpm under sterile regular saline irrigation. An osteotomy was performed with an 8-mm trephine drill to create cortical bone defects. After that, the implant recipient sites were prepared in the defect center according to the manufacturer’s recommendations. The implants were inserted into the drilled holes with bone engagement in the apical portion to obtain primary implant stability (Figure 4A).

Approximately 2-mm circumferential peri-implant bone defects were created, which were filled with the following biomaterials: (1) Algipore, (2) Algipore+BMSCs (2 × 10^6^ cells per 40 mg of scaffold), (3) Biphasic, and (4) Biphasic+BMSCs (2 × 10^6^ cells per 40 mg of scaffold) (*n* = 5) (Figure 4F). BMSCs (2.0 × 10^6^) in 0.5 mL of PBS were homogeneously mixed with Algipore and Biphasic (40 mg) and incubated for additional 4–6 h to allow cell attachment before implantation in peri-implant bone defects. Finally, a prehydrated collagen membrane (20 mm × 30 mm; Symbios Collagen Membrane) was placed to cover the implants and defect regions (Figure 4G). The tissue was repositioned and sutured with bioresorbable sutures (Vicryl 4.0, Ethicon, Somerville, NJ, USA) and nylon 5.0 sutures (Unik Surgical Sutures MRG, Taipei, Taiwan). Postoperatively, 5.0 mg/kg antibiotics (Baytril, Bayer, Leverkusen, Germany) and 4.0 mg/kg analgesics (Rimadyl, Pfizer, New York, USA) were prescribed for three days for infection prevention and pain control. All experimental animals were euthanized via CO_2_ overdose after 4 or 8 weeks of healing.

### 4.5. Measurement of Implant Stability Parameters

Implant stability parameters were assessed immediately after implant insertion (0 weeks) and after 4 and 8 weeks of healing (Figure 4B–E,I,J). Implant stability parameters, including implant stability quotient (ISQ) and Periotest value (PTV) values, were measured using Penguin (Integration Diagnostics Sweden AB, Göteborg, Sweden) and Periotest M device (Siemens AG, Bensheim, Germany) devices, as described previously [2,40].

Briefly, for the measurement of ISQ, an aluminum rod with a magnet called a “SmartPeg” (type 1) (Integration Diagnostics Sweden AB, Göteborg, Sweden) was screwed into the dental implant and tightened to approximately 5 N-cm. Next, the transducer probe was maintained perpendicular to the long axis of the SmartPeg to standardize the experimental procedure until the instrument beeped and displayed the ISQ value (Figure 4B). The resonance frequency of the implant was measured using the Penguin device and automatically converted to an ISQ value, ranging from 0 to 100, with higher values indicating higher implant stability (Figure 4C,I). Subsequently, the healing abutment was screwed into the implants for measuring the PTV values by using the Periotest device (Figure 4D). In brief, the impact rod strikes the healing abutment of the implants, and the contact time during detection is automatically calculated as PTV values, ranging from −8 to +50, with lower values indicating higher implant stability (Figure 4E,J).

### 4.6. Micro-Computed Tomography Measurement

Micro-computed tomography (micro-CT) analysis of bone regeneration at the implant–bone interface was performed as described in our previous studies [2,40,41,42]. After the dissection of the tibia, the whole bone blocks containing the implants were removed and fixed in 10% neutral buffered formalin. The samples of the implant–bone assembly were scanned using micro-CT (Skyscan 1076, Skyscan, Antwerp, Belgium) at a voltage of 50 kV, an electric current of 100 mA, and a pixel resolution of 18 μm with a 0.5-mm aluminum filter. After that, three-dimensional (3D) image models were reconstructed and analyzed using CT-Analyser software (version 3) (CTAn, Skyscan). The volume of interest (VOI) was selected around the implant surface and defined as the specific area within a 500-μm expanded outline of the implant over the entire thread portion of the implant. Optimal thresholds were selected and determined by segmenting the micro-CT images to discriminate the newly formed bone from the connective tissue and grafting materials. Finally, the percentage of bone volume (BV) to the total tissue volume (TV) within the VOI (BV/TV%; BV density) was determined.

### 4.7. Histology and Histomorphometric Analysis

After the micro-CT scan, the whole bone blocks containing implants and bone grafting materials were prepared for non-decalcified histological processing according to our previous studies [2,40]. Briefly, the entire bone blocks containing implants and bone grafting materials were dehydrated with a series of grades of ethanol xylene, infiltrated, and embedded in methylmethacrylate resin (Technovit 9100 NEU; Heraeus Kulzer, Wehrheim, Germany) according to the manufacturer’s instructions. After polymerization, each resin-embedded specimen was cut into approximately 300-μm-thick sections using a diamond band saw (AZCL40, Yeong Shin Hardware, Taipei, Taiwan). Through grounding and polishing the thick sections, the sections were finished to a final thickness of approximately 50 μm and stained with 1% toluidine blue (Sigma–Aldrich Chemie GmbH, Buchs, Switzerland) for 20 min. The images of the histological slides were examined and acquired using a standard light microscope (Leica DM500, Leica Microsystems, Wetzlar, Germany) equipped with a SPOT digital camera (Diagnostic Instruments, Sterling Heights, MI, USA). As previously described [2,40], the total bone–implant contact (BIC) percentage was calculated by measuring the amount of mineralized bone directly in contact with the implant surface over the entire length of the implant by using an Image-Pro Plus 6.0 image analysis system (Media Cybernetics, Silver Spring, MD, USA) at 40× magnification.

### 4.8. Statistical Analysis

SPSS (version 19, SPSS, Chicago, IL, USA) was used for all statistical analyses. All data, including ISQ, DF, BV/TV, and BIC values, are expressed as mean ± standard deviation. Comparisons were made using a one-way analysis of variance with Tukey’s honest significant difference test, and *p* < 0.05 was set as substantial.

## 5. Conclusions

Our results demonstrated that incorporating Algipore and Biphasic BGMs with BMSCs can synergistically and efficiently promote bone regeneration and osseointegration in severe peri-implant bone defects. Furthermore, Algipore BGM incorporation with BSMCs demonstrated good biomechanical performance in enhancing dental implant stability and osseointegration potential.

## Data Availability

The data are available from the corresponding author upon reasonable request.
